# 

**Published:** 1860-11

**Authors:** 


☞ Clinical instruction in Bellevue Hospital, New York, has, by order of the Commissioners, been made free to students, almost at the same time that a similar course was adopted by the Board of the Philadelphia Hospital. This makes all the Hospitals of New York accessible to students free of charge. It is, we think, high time that the Pennsylvania Hospital should follow.

### List of Payments.

M. Woodruff, Ga., $3; Dr. J. J. Cooper, Ga., $6; Dr. J. C. Respess, Ga., $6; G. L. Hunter, S. C., $2; W. S. Brooks, Ga., $2; D. M. Murray, Ga., $6; J. A. Eve, Ga., $6; J. G. Broyles, Miss., $10; J. M. Couch, Ga., $5; J. W. Bailey, Ga., $2; W. B. Ayres, Ga., $3, J. T. McKey, Ga., $3; J. H. Parrish, Ala., $14; L. M. Hook, Ga., $2; Wm. H. Moore, N. C., $6; G. W. Powell, Ala., $3; A. T. Henry, Ga., $3; W. Gordon, Ga., $6; A. S. Fowler, $12; E. C. Mayer, Ga., $9; J. T. Wamock, Ala., $4.75; W. B. Sikes, Ga., $3; S. S. E. Bell, Ala., $5; W. D. Cunningham, Ga., $2; Wm. D. Prior, Ala., $3; J. C. Nott, Ala., $15; P. C. Sadler, Texas, $10; Jeremiah Hilsman, Ga., $3; E. M. Brown, S. C., $2; F. M. Hunt, Ga., $3; J. C. Turnipseed, Ga., $3; J. F. M. Davis, Ga., $2; M. A. Gaston, Ga., $2; G. L. Jones, Ga., $9; S. P. Hobgood, Ga., $6; M. F. Siddell, Ga., $10; T. R. Ashford, Ga., $3; R. A. T. Ridley, Ga., $15; J. S. Sappington, Ala., $5; G. W. Barnett, S. C., $2.

### TO CORRESPONDENTS.

By a recent change in the management of the Journal, its whole business affairs will hereafter come under the supervision of the Editor, and it is therefore requested that all Communications, whether relating to the Editorial, Subscription, Advertising or Financial Departments, should be addressed to J. G. Westmoreland, or Editor of Medical and Surgical Journal, Atlanta, Ga.

